# Clonal diversity and spatial genetic structure in the long-lived herb, Prairie trillium

**DOI:** 10.1371/journal.pone.0224123

**Published:** 2019-10-21

**Authors:** Jennifer R. Mandel, C. Kendall Major, Randall J. Bayer, James E. Moore

**Affiliations:** 1 Department of Biological Sciences, The University of Memphis, Memphis, TN, United States of America; 2 Center for Biodiversity, The University of Memphis, Memphis, TN, United States of America; 3 Edward J. Meeman Biological Station, Millington, TN, United States of America; 4 Department of Biology, Christian Brothers University, Memphis, TN, United States of America; Brigham Young University, UNITED STATES

## Abstract

Combining population genetic studies with demographic surveys in long-lived clonal herbs can yield insight into the population dynamics of clonal plant populations. In this study, we assayed clonal diversity and spatial genetic structure in a population of a long-lived understory herb, *Trillium recurvatum*, that has been the focus of a demographic study spanning 26 years at the Meeman Biological Station in Memphis, Tennessee, USA. Using a set of five newly developed simple sequence repeat markers first reported here, we assessed 1) the extent of clonal diversity within the Meeman site, 2) the degree to which genetic diversity varies with stage class (juvenile, non-flowering, and flowering adults) at this site, 3) whether there is spatial genetic structure at the Meeman site, and 4) how measures of genetic diversity and inbreeding at the Meeman site compare to two additional nearby populations. Along with these analyses, we calculated and compared traditional population genetic metrics with information theory-based diversity indices. Although clonal propagation was present, the focal population displayed moderate levels of clonal diversity comprising 81 genets from the 174 individuals sampled. In the focal site, we also found that genetic diversity was highest in the flowering stage class when compared to the non-flowering and juvenile classes. We report that genets exhibited spatial genetic structure in the focal site exhibiting values for the *Sp* statistic of 0.00199 for linear distance and 0.0271 for log distance. Measures of unbiased gene diversity and the inbreeding coefficient were comparable across the sampled populations. Our results provide complementary genetic data to previous demographic studies in *T*. *recurvatum*, and these findings provide data for future studies aimed at integrating the degree of clonality, genetic variation, and population dynamics in this species. Our findings suggest that *T*. *recurvatum* at the focal Meeman site displays higher levels of sexual reproduction than were previously suggested, and spatial genetic structure estimates were comparable to other plant species with mixed and outcrossing mating strategies.

## Introduction

Clonal plants comprise at least 40% of our planet’s flora [1] and can dominate many environments [2]. Although there are costs, such as ease of disease transmission and reduced available mate sources for sexual reproduction [3–5], clonal growth has many advantages. These advantages include allowing for more extensive nutrient acquisition, the accumulation of greater biomass, and minimizing the mortality risk of the genet [6]. Thus, the genets of a clonal species may experience a long life span potentially mitigating the loss of genetic variation over time [7]. Still, clonal reproduction limits the potential for recombination to generate genotypic novelty. The ability to combine sexual reproduction and clonal growth can be beneficial when pollination success is low [8] or when local resource abundance is heterogeneous [9]. Silvertown [10] suggested that sexual reproduction may be favored over clonal propagation in disturbed habitats. Conversely clonal propagation may be advantageous when sexual fecundity is low (such as in small populations or when self-incompatibility is present) or when genets are colonizing new environments [10]. Therefore, the combination of sexual reproduction and clonal growth may be beneficial for maintaining population viability especially in environments that are unpredictable or have high variability [11].

Studies investigating population dynamics in clonal plant species, e.g., estimating population size, stage structure, and population growth, benefit from knowing the extent of clonality and number of unique genotypes present since these measures can inform mating system and genetic diversity within a population. Estimates of genetic population size (i.e., number of genets) can inform the degree to which a population combines sexual reproduction and clonal growth. The genetic composition of any given population depends on a variety of factors including environmental conditions, breeding strategy, pollination and dispersal biology, and competition, and may fluctuate over time [10, 12–13]. The balance of sexual reproduction and clonality can be estimated by identifying the composition of the number of genets and ramets. For example, if a clonal population relies more heavily on sexual reproduction, the population may harbor considerable numbers of unique genets. Conversely, a population could comprise only a few successful genets that dominate the site leading to lower numbers of unique genotypes. In addition, the knowledge of clonal diversity and the spatial structuring of genets in a population can yield insights into the mating system, growth form, and patterns of pollen and seed dispersal [5,14,15].

Often though, accurate estimates of the genetic population size are difficult in plant species that propagate clonally because genets are not easily distinguished from ramets. Invasive sampling techniques, such as unearthing rhizomes, may be required to determine the extent of clonality; however, genetic markers, when available, can be used in a less destructive manner. Genetic markers can be used to estimate the number of unique genotypes and the extent of clonality, as well as, overall levels of population genetic diversity. The goal for the current study was to understand the extent of clonality and levels of genetic diversity in a focal population of a clonal forest understory herb, *Trillium recurvatum* Beck. This species is self-incompatible [16] and has been the subject of a long-term population dynamics and viability study [17].

In a study spanning more than 20 years (9 years of data collected), Moore et al. [17] analysed demographic data from a focal population of *T*. *recurvatum* found within the center of the species’ range. The population demographic structure in *T*. *recurvatum* reflects clonal growth and rates of transition from young (juvenile) to adult non-flowering to flowering stage class [17]. In *T*. *recurvatum* as in other *Trillium* species, ramets can regress from a flowering to non-flowering individual. These ‘backward’ transitions can be affected by biotic factors (e.g. deer herbivory) that cause ramets to regress in stage, have lower fecundity, and have lower probability of producing new ramets [18–19].

Moore et al. [17] found that finite growth (λ) increased over all age classes, with non-flowering ramets showing the highest rate of increase. That focal population is also marked by ‘booms’ and ‘busts’ in the number of flowering individuals, which likely indicates a high degree of regression from flowering to non-flowering ramets across years. Regressions from flowering to non-flowering individuals may influence rates of sexual reproduction and genetic composition, especially in this self-incompatible species in which the frequency of selfing (and thus non-productive matings) can be related to the size and spatial distribution of genets within the population (e.g., [5]). Regressions could be particularly important in small and isolated populations where genetic drift would reduce the number of reproductive individuals thus reducing mating opportunities [20]. While the focal *T*. *recurvatum* population has been intensely studied for more than 20 years for demography and population dynamics, little information about the number of genetic individuals, degree of clonality, or spatial structure of genets in the population was known. In this study, we asked four questions: 1) what is the extent of clonality in the Meeman focal population? 2) does genetic variation vary within stage class of the ramet (e.g., juvenile, non-flowering, and flowering)? 3) what is the spatial genetic structure in the focal population? 4) in general terms, how does the genetic variation and inbreeding in the Meeman focal population compare to nearby *T*. *recurvatum* populations? We developed and report here five new simple sequence repeat (SSR, microsatellite) markers for *T*. *recurvatum* and extensively sampled this population to address these questions.

## Methods

### Species biology

*Trillium recurvatum* Beck. is a sessile-flowered rhizotomous perennial understory herb with northern limits in southern Wisconsin and Michigan, west to eastern Missouri and Iowa, extending east to Pennsylvania, and south into northern Alabama and Louisiana. The species reproduces both sexually through seed and asexually through rhizomes (clonal) and is self-incompatible [21]. At the edge of the *T*. *recurvatum* geographic distribution, it is considered rare (e.g., rare in the state of Alabama and a species of special concern in Wisconsin). In Tennessee, *T*. *recurvatum* occurs primarily west of the Cumberland Plateau. In our study area in West Tennessee, plants emerge in mid-to-late February, flower in late March to early April and senesce by the end of April or early May when woody overstory leafburst occurs. Similar to other *Trillium* species, ants are likely the primary dispersal agent [22]. When sexual reproduction occurs, seeds exhibit double dormancy and ants collect the seeds and feed on elaiosomes, discarding seeds in tunnels until germination occurs [23].

### Study sites

Edward J. Meeman Biological Station (MBS) (owned and operated by the University of Memphis) is a 252 ha site ~ 40 Km north of Memphis, TN (Shelby County), and 3 Km east of the Mississippi River on the third Chickasaw Bluff. The primary soil type is Memphis silt loams composed of loess, which have high erodibility on slopes in this region [24]. The yearly average temperature is 16°C. July and January are the warmest and coldest months, respectively (26.6°C and 4°C). Average yearly precipitation is 1320 mm primarily occurring during the winter and spring months. In early spring, the only other major ground layer constituent at this site is *Podophyllum peltatum* L. This *Trillium* site was sampled from a west-facing slope. In addition to the sampling at MBS, we also sampled *T*. *recurvatum* from two populations occurring in southwestern Shelby County (SC1 and SC2 populations) roughly 30 Km from the Meeman site in order to provide a comparison for population genetic variation. As much of the land in Shelby County is urban, we chose these sites as they are (to the best of our knowledge) natural, and undisturbed populations of *T*. *recurvatum*. Permission to collect leaves was obtained and this study did not involve endangered or protected species.

### Plant collection

In the Meeman site, the species tends to grow in both distinct clusters and as dispersed ramets. Leaf samples of *T*. *recurvatum* collected from the Meeman population in early Spring of 2014 were sampled using a standardized transect array consisting of 25 2-by-2 meter (m) quadrats. This five-by-five array (25 total quadrats) was established in 1990 along a west-facing slope at MBS and is the same array reported in Moore et al. [17]. Columns of the array are labelled A, B, C, D, and E; rows are numbered, 1, 2, 3, 4, and 5 (e.g., the first row and column quadrat is A1). Quadrats were separated by a minimum of two meters. Up to five plants were sampled from as many spatially distinct clusters (separated by at least 0.2 m) as possible within each quadrat. All plants sampled were recorded for their stage class: juvenile (one-leaf individual, representing juvenile ramets or seedlings), non-flowering adult (three-leaf individual), and flowering adult (flowering three-leaf individual). Leaf samples were also collected from two sites in southwestern Shelby County (13 individuals from SC1 and 20 individuals SC2 populations); the goal of the SC sites sampling was not to evaluate clonality but to provide a comparison for the levels of genetic variability found at Meeman. Unlike the Meeman site where we sampled up to five plants per cluster, here, we only sampled leaves from individuals that were more than 2 m apart in order to increase the likelihood of sampling from distinct genetic individuals in these sites.

### Genetic marker analysis

Genomic DNA was isolated from a 2 cm portion of leaf tissue obtained from all individual leaves using the SP Omega Biotek Plant Kit. DNA was quantified and quality assayed using a Nanodrop spectrophotometer (Thermo Scientific). Each sample was standardized to 20 ng/μl according to the Nanodrop readings. Five Simple Sequence Repeat (SSRs or microsatellite) markers were utilized for this study: TR-6, TR-7, TR-13, TR-15, and TR-17 (S1 Table contains data details of primer selection, as well as, PCR conditions). In order to identify microsatellite loci, a repeat enrichment of short tandem repeats followed by next-generation sequencing approach was used. The enrichment and sequencing were performed by the Evolutionary Genetics Core Facility at Cornell University following a modified protocol (see [25] for additional detail including reagent recipes) which employs streptavidin beads and biotinylated microsatellite probes. Microsatellite loci were identified by enriching an Illumina prepared genomic library of *T*. *recurvatum* (two samples pooled from the Meeman site) for short repeats (i.e., GT, TC, TTC, GTA, TTTC, etc.). Enrichment of repeats was critical for developing microsatellites since the genome of *T*. *recurvatum* is extremely large for a plant species at ~ 50Gb [26]. The enriched library was sequenced at the Cornell Life sciences Core Laboratory Center for 2 x 250 paired end sequencing on an Illumina MiSeq. Reads were quality trimmed and assembled *de novo* into contigs using SeqMan NGen v11 (https://www.dnastar.com/t-nextgen-seqman-ngen.aspx), and microsatellite loci and primer pairs were identified with msatcommander 1.0.3 [27] using default settings enabling the "design primers" function and the “repeats” and “primers” options for the output files (for Mac OSX).

PCR amplification of the five loci used in the study was carried out in a reaction consisting of 10X PCR buffer (100mM KCl, 100mM Tris HCl (pH9.0), 80mM (NH4)2SO4, and 1.0% Triton X-100), MgCl2 (25mM), dNTPs (each at 20mM), forward primer (5μM), reverse primer (20μM), fluorescently labelled M13 primer (10μM) [28], Taq DNA polymerase, and 1 μl template DNA (20 ng/μl). Amounts and concentrations were optimized for each locus. The PCR conditions followed a general “touchdown” program varied per locus see S1 Table: 3 min at 95°C; 10 cycles of 30 s at 94°C, 30 s at (X)°C and 45 s or 1 min at 72°C, annealing temperature decreasing to (X-10)°C by 1°C per cycle, followed by 30 cycles of 30 s at 94°C, 30 s at (X-10)°C, 45 s or 1 min at 72°C, followed by 10 min at 72°C. The PCR amplicons were diluted 1:10 and visualized using an Applied Biosystems 3130xl DNA sequencer with GeneScan 500 LIZ dye Size Standard (Applied Biosystems) included in each lane to allow for accurate fragment size determination. Alleles were scored using the software package GeneMarker v. 2.6.3 (SoftGenetics), and two people scored and concurred on all sample calls (CKM and JRM). Evidence for null alleles, large allele dropout, and stuttering was assessed using MICRO-CHECKER version 2.2.3 [29].

### Clonal and genetic diversity analyses including stage class comparisons

We sampled 174 plants in the Meeman population; however, some of these samples could represent ramets of the same genet and potentially bias genetic diversity statistics. Following Mandel [30] and Bentley and Mauricio [31], we therefore analysed our data set in two ways: 1) in the first, we assessed genetic diversity on the *full* data set (all 174 samples included), and 2) in the second, we analysed genetic diversity on a *genet-only* data set (unique multilocus genotypes). In order to identify the *genet-only* data set, we used the software program GENODIVE v. 2.0b27 [32] to assign individuals to multilocus genets (or clones). Assignment to genets was carried out using the Meirmans and Van Tienderen [32] algorithm which calculates a genetic distance matrix and uses a *clonal threshold*. This information is used to delineate plants as unique multilocus genotypes (genets vs. ramets) using the stepwise mutation model option and missing data coded as one mutational step. When the genetic distance between two individuals falls below the threshold (set to 2 for our study based upon recommendations by the authors of the programs), the two individuals are assigned to the same genet. For the *full* data set (174 individuals), we assessed genetic diversity both based on spatial organization in 2 m X 2 m quadrats (up to 25) and by stage class of the sample (juvenile, non-flowering adult, or flowering adult). Using GENODIVE, we calculated the number of unique multilocus genotypes, effective number of multilocus genotypes based on rarefaction, Simpson’s Diversity, evenness and the Shannon-Weiner index corrected for sample size. Bootstrap tests (1000 replicates) for differences in clonal diversity between pairs of stage classes were also performed in GENODIVE. In GenAlEx v. 6.5 [33], we calculated Nei's unbiased gene diversity corrected for sample size (uHe) for all groupings. Recent work in the crop-wild gene flow literature (e.g., [34–35]) and more broadly in population genetics [36], has demonstrated that information theory-based measures (i.e., Shannon-Weiner) may be more sensitive to detecting genetic diversity differences among groups than traditional population genetic measures. We therefore compared these measures (uHe vs. uS-W) in our data set here.

Genetic analysis of the *genet-only* data set (unique multilocus genotypes only, G from above, i.e., clones removed) consisted of measures of genetic diversity and genetic distance using GenAlEx. Note that from the set of multilocus genotypes, we chose the sample with the least missing data to represent that multilocus genotype. Observed heterozygosity Ho, Nei’s [37] unbiased nuclear gene diversity uHe, the Shannon information index (H), and the inbreeding coefficient (F) averaged across loci per population were calculated in GenAlEx.

A genotype accumulation curve was used to determine the minimum number of loci needed to discriminate between individuals (poppr::gac; 10,000 permutations; [38, 39]). The probability that that identical genotypes were not obtained by chance was calculated using a multilocus probability for codominant genotypes, Pcgen = (∏*pi*)2^h^, where *pi* is the frequency for each allele observed in the multilocus genotype and h is the number of heterozygous loci. The probability of obtaining n—1 more copies of a particular clonal genotype by chance alone is given by (Pcgen)^n—1^, where n is the number of times the genotype was observed in the population.

### Spatial genetic structure in the focal Meeman Site

A test for genetic isolation-by-distance (IBD) was performed using a Mantel test for matrix correspondence [40] on all 174 individuals in the Meeman population between a spatial distance matrix (all pairwise spatial distances in meters) and a genetic distance matrix (all pairwise Nei’s [37] genetic distances) using GenAlEx [33]. Matrix correspondence between the geographic matrix and the Nei’s genetic distance matrix was tested using GenAlEx. Three additional Mantel tests were performed by separating the dataset into stage classes, i.e., juvenile, non-flowering, and flowering. In an effort to visualize the spatial distribution of genets, we also created an occupancy table depicting all unique genotypes in the 5 x 5 array. This model was generated using R v 3.3.2 [39] with the following packages: reshape2; ggplot2; scales; and plyr. A more detailed approach for estimating spatial genetic structure (as compared to the Mantel method which tests for correlations between matrices) was also carried out by estimating the *Sp* statistic which incorporates pairwise kinship coefficients and linear or log distances into the calculations. Estimates of the *Sp* statistic (a metric to both quantify and compare spatial genetic structure and is related to the mating system being higher in selfing species) were obtained for the Meeman sites using *b*, the slope of the regression, of F*ij* on linear and log distance and the mean pairwise kinship coefficient with the formula *Sp* = -*b* (1- *F1*) where *F1* is the first distance class following [15] in SPAGeDi version 1.5 [41]. Tests of 1000 random permutations were carried out to assess whether the kinship coefficient and regression slopes *b* were significantly different from zero.

### Comparison to nearby populations

As a comparison of genetic diversity measures with the Meeman population, we also sampled 13 and 20 plants from two locations, respectively, in southwestern Shelby County, Tennessee, USA. Even though we attempted to collect in a way in which we maximized unique genetic individuals, we still analyzed the data in the same manner as for the 174 individuals from Meeman. Therefore, for these samples, we also assigned genets (clones) in GENODIVE using the same parameters as above and making genets specific to each population. For the *full* data set (13 and 20 individuals), we calculated the number of unique multilocus genotypes (G), effective number of multilocus genotypes based on rarefaction (effG), Simpson’s Diversity (D), evenness (E) and the Shannon-Weiner index corrected for sample size (uS-W). We also calculated genotypic richness (R) per site as (G− 1) / (N− 1) where G is the number of genets and N is the number of samples. In GenAlEx, we also calculated Nei's unbiased gene diversity corrected for sample size (uHe). For the *genet-only* data set (retaining only unique genotypes as in the Meeman population), we calculated observed heterozygosity Ho, Nei’s [37] unbiased nuclear gene diversity uHe, the Shannon information index H, and the inbreeding coefficient (F). Using GenAlEx, we also calculated F_ST_ = (H_T_- Mean H_S_) / H_T_ where H_T_ describes the expected heterozygosity if all populations were treated as one, and Mean H_S_ describes the average of the within population expected heterozygosity across populations. We calculated F_ST_ among all three sites and between the SC1 and SC2 separately. Genetic distances amongst genets from Meeman and the SC1 and SC2 populations were investigated graphically using Principal Coordinate Analysis (PCoA). For this, a standard genetic distance matrix was constructed based on the nuclear multi-locus genotypes, the resulting matrix was used for the PCoA, and the first two principal coordinates were graphed in 2-dimensional space.

## Results

### Clonal diversity in the focal Meeman site

Evidence for null alleles, large allele dropout, and stuttering was assessed and the potential for null alleles or stuttering was suggested at Locus 6 and Locus 17; however, the results were not consistent across the populations (S2 Table). The genotype accumulation curve demonstrated five loci was near the cut-off for discriminating individuals (S1 Fig), and the calculations for Pcgen provided additional support for the discriminatory power of the loci. A total of 81 genets were identified from 174 individuals (using the Assign clones function of GenoDive). For the 81 genets at the Meeman site, all but five of these values for Pcgen fell below the 0.05 threshold with three of these being 0.059. Therefore, despite the use of only five SSRs, the ability to distinguish genotypes was high with only two genotypes having low exclusion power (genet/clone 68 and 75, 0.1647 and 0.1807 respectively). Additionally, for the 81 genets at the Meeman site where multiple ramets were observed, (Pcgen)^n—1^ values ranged from 0.0056 to 3.5E-63. Thus, the statistical probability that sampled plants with the same multilocus genotype were different genetic individuals was low and below the 0.05 threshold for all 81 genotypes. Table 1 gives the population genetic data for the three sites and S3 Table gives by locus.

**Table 1:** Genetic diversity statistics of the *genet* data set for three populations.

Population	N_a_	N_e_	H_o_	H_e_	uH_e_
Meeman	174	81	35.0	0.98	0.43
SC1	13	13	13.0	1	1
SC2	20	18	16.7	0.99	0.93

The number of different alleles, (N_a_); number of effective alleles (N_e_); observed heterozygosity (H_o_); expected heterozygosity (H_e_); Nei's unbiased expected heterozygosity (uH_e_).

### Genetic variation of three stage classes

Among the three stage classes (Table 2), the flowering class demonstrated the highest clonal diversity values for effective number of multilocus genotypes, Simpson’s Diversity, Evenness, the Shannon-Weiner Index, and gene diversity (though non-flowering individuals were identical for gene diversity). In order to assess whether the flowering stage class demonstrated statistically different levels of diversity, we used a 1000 bootstrap replicates test, the uS-W (Shannon-Diversity) was significantly higher in the flowering class when compared to non-flowering (1.87 vs. 1.72, p = 0.029) and juvenile classes (1.87 vs. 1.70, p = 0.039) (Table 2).

**Table 2:** Genetic diversity of the three stage classes.

Stage Class	N	G	R	effG	D	E	uS-W	uHe
Juvenile	49	31	0.63	22.0	0.97	0.71	1.70^a^	0.49
Non-flowering	69	39	0.56	20.0	0.96	0.511	1.72^a^	0.51
Flowering	56	39	0.70	30.2	0.98	0.77	1.87^b^	0.51

The number of sampled individuals, N; number of unique multilocus genotypes (G); genotypic richness (R); effective number of multilocus genotypes based on rarefaction (effG); Simpson’s Diversity (D); evenness (E); the Shannon-Weiner index corrected for sample size (uS-W); significance as determined from 1000 bootstrap replicates is shown with corresponding subscript symbols a and b; Nei's unbiased gene diversity corrected for sample size (uHe).

### Spatial genetic structure

**Fig 1:**
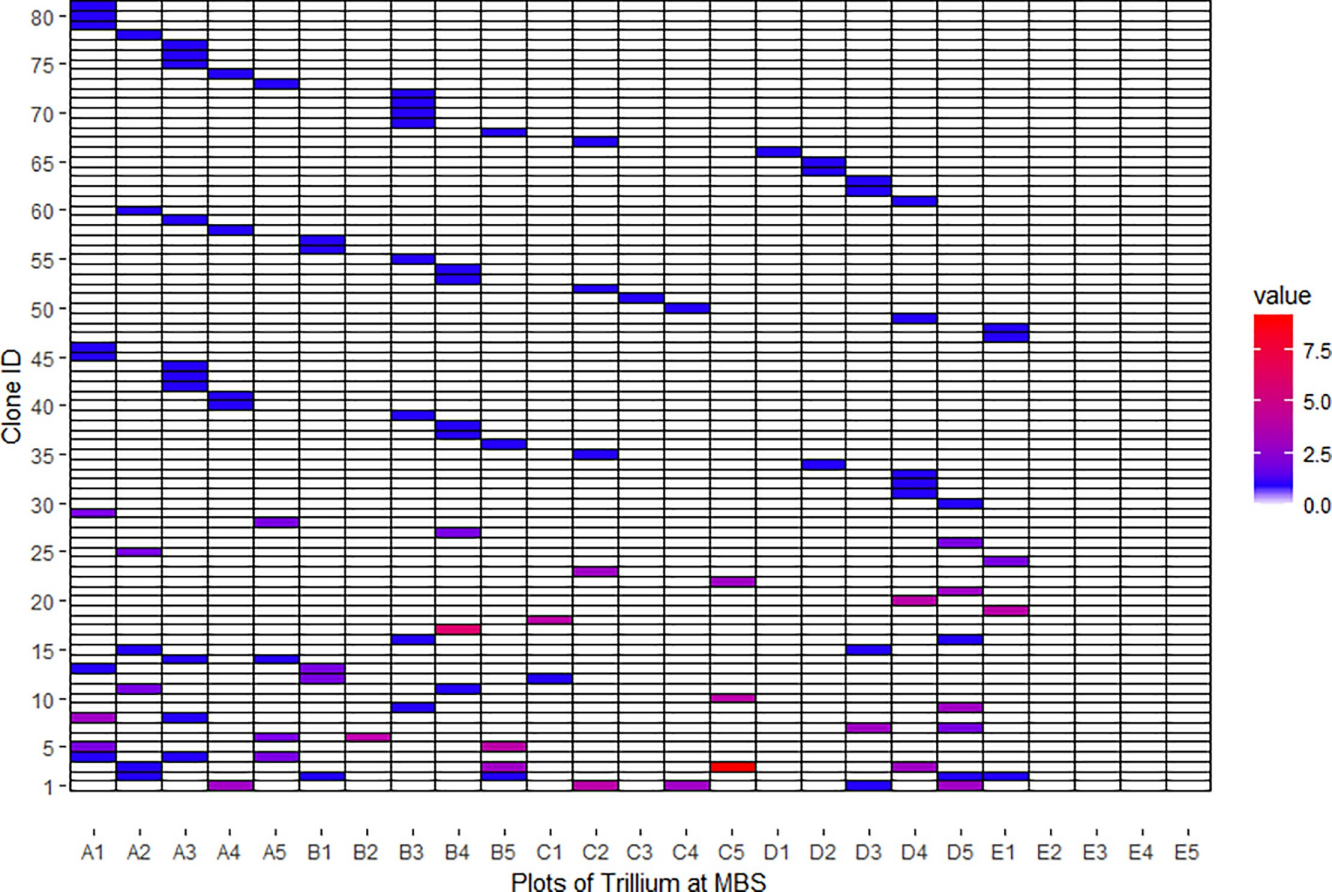
Occupancy (in each quadrat) table for all 81 genet/clones. The color of the block is representative of the number of plants sampled for that genet, as well as, an inset for graphical description of the 5 by 5 transect array.

**Fig 2:**
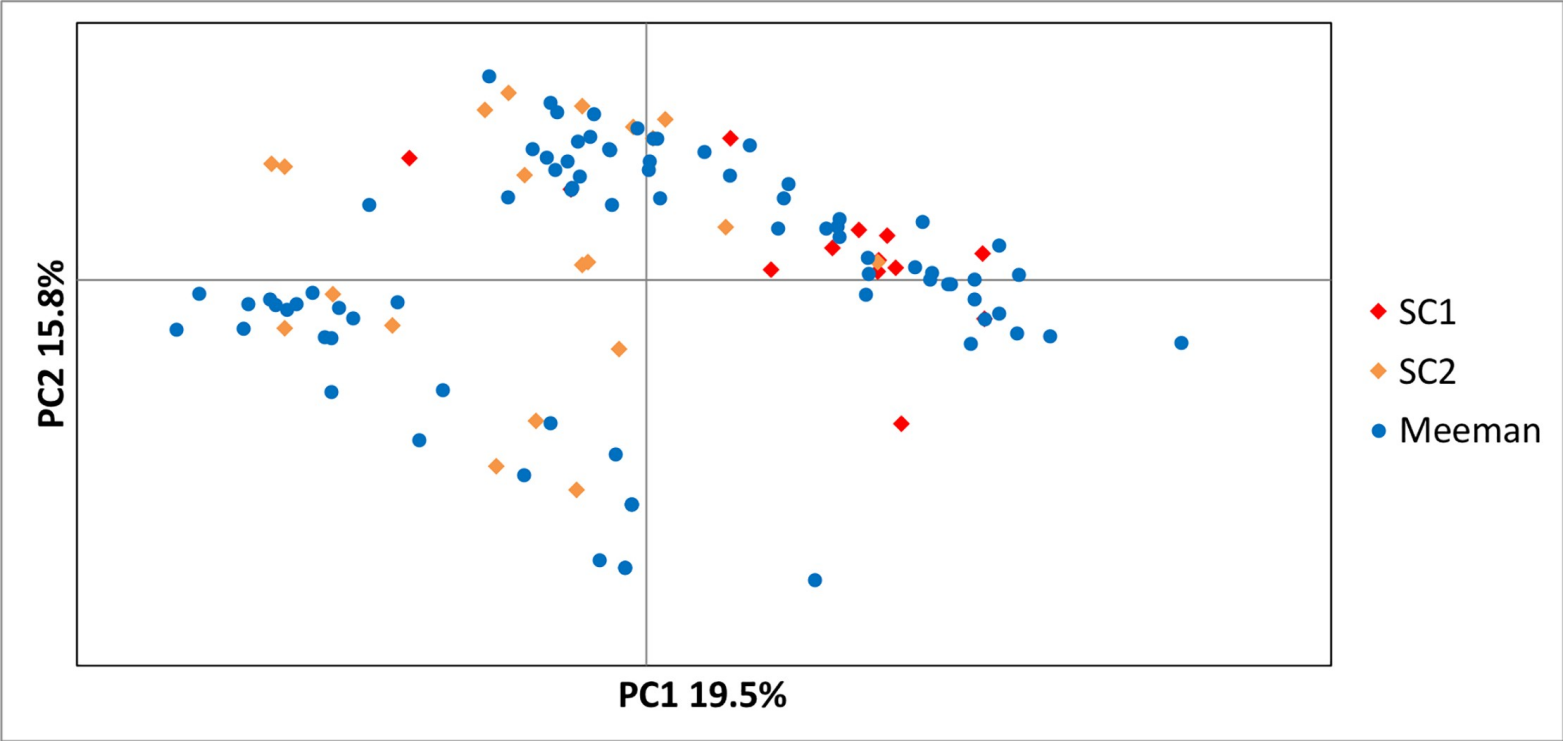
Principal coordinates analysis. The first two principal coordinates graphed in 2-dimensional space for the three genet-only data set populations: Meeman, Shelby County 1 and 2.

In order to represent clonal diversity graphically, Fig 1 provides an occupancy (in each quadrat) table for all 81 genets and the color of the block is representative of the number of plants sampled for that genet. Inspection of this figure demonstrates how some genets are shared across quadrats, as well as, how frequent particular genets were in the site. S4 Table provides the raw location data and stage class for each genet, S5 Table provides the raw data for Fig 1, and S6 Table provides the data for the microsatellite locus genotypes. Within the Meeman site, genets were differentiated along PC2 though this did not correspond to any spatial structure along the hillside where we collected (Fig 2; PC1 19.5%, PC2 15.8%). All four (juvenile, non-flowering adult, flowering adult, and total) Mantel tests for matrix correspondence between genetic and geographic distance were non-significant (R^2^ = 0.0006, p = 0.06; R^2^ = 0.0015, p = 0.22; R^2^ = 0.0004, p = 0.33; R^2^ = 0.003, p = 0.28). However, the results of the spatial genetic structure analyses as assessed by the *Sp* statistics estimated values of 0.00199 for linear distance and 0.0271 for log distance, and permutation tests showed that the kinship coefficient (0.192) and regression slopes *b*, which were negative (-0.00248 for linear and -0.0338 for log distance), were significantly different from zero in all cases.

### Comparison to nearby populations

Diversity statistics for the *full* data sets (not taking into account clonality) demonstrated lower values in the focal Meeman population as compared to the other Shelby County populations surveyed as measured by the effective number of multilocus genotypes, Simpson’s Diversity, Evenness, the Shannon-Weiner Index, and gene diversity (Table 3). Note, that the unbiased measure uS-W cannot be calculated when all samples have different genotypes as in SC1. When genets-only data sets were considered, the Meeman site showed slightly higher values (though not statistically significant) for observed heterozygosity, unbiased gene diversity, and the Shannon information index though these values were all quite comparable. In terms of inbreeding, the Meeman site had slightly lower levels (though not statistically significant) of inbreeding though again all values were comparable (Table 4, S3 Table). The PCoA in general revealed overlap amongst sampled individuals across the three sites indicating low levels of genetic differentiation among sites. In agreement with this low genetic differentiation, the F_ST_ among all three sites was 0.047 (p < 0.05). The two southwestern Shelby County populations also mostly overlapped but showed some differentiation along PC1, and F_ST_ between these two sites was similarly low: F_ST_ 0.041 (p < 0.05).

**Table 3:** Clonal diversity statistics of the *full* data set for three populations.

Population	N	G	R	effG	D	E	uS-W
Meeman	174	81	0.46	35.0	0.98	0.43	1.95
SC1	13	13	1	13.0	1	1	---
SC2	20	18	0.89	16.7	0.99	0.93	1.96

The number of sampled individuals, (N); number of unique multilocus genotypes (G); genotypic richness (R); effective number of multilocus genotypes based on rarefaction (effG); Simpson’s Diversity (D); evenness (E); the Shannon-Weiner index corrected for sample size (uS-W); Nei's unbiased gene diversity corrected for sample size (uHe).

**Table 4:** Genetic diversity statistics for three *genet-only* populations.

Population	G	Ho	uHe	Shannon H	F
Meeman	81	0.42 (0.12)	0.54 (0.09)	1.01 (0.20)	0.22 (0.16)
SC1	13	0.30 (0.10)	0.49 (0.08)	0.79 (0.13)	0.28 (0.22)
SC2	18	0.26 (0.09)	0.55 (0.07)	0.99 (0.18)	0.49 (0.18)

The number of unique multilocus genotypes (G); observed heterozygosity (Ho); Nei's unbiased gene diversity corrected for sample size (uHe); the Shannon information index (H), the inbreeding coefficient (F) averaged across loci per population. Standard errors are shown in parentheses.

## Discussion

The measures of both clonal and genetic diversity in the focal Meeman site suggested that the populations consisted of a mixture of both asexual and sexual reproduction with roughly half of the sampled plants representing unique genotypes (81 genets out of 174 sampled) and a mean unbiased gene diversity of 0.54. Moreover, the inbreeding coefficient, as measured here, was positive which is in contrast to other findings in highly clonal species where values are often highly negative [42]. Moore et al. [17] reported that this population of *T*. *recurvatum* primarily reproduced vegetatively as only 11% of stems originated from seed. The population genetic data we present here demonstrates that more sexual reproduction is occurring than was previously thought. Indeed, our finding that *T*. *recurvatum* harbors moderate levels of genetic diversity is in agreement with population genetic studies in other clonal species (e.g., [30; 43]). Moreover, Gonzales et al. [44] found high levels of clonal diversity in the related *T*. *cuneatum* and noted differences in clonal reproduction in Piedmont versus mountain populations in the Southeast United States. However, Gonzales and Hamrick [45] investigated clonal and genetic diversity in *T*. *reliquum* and found low levels of genetic diversity that they attributed to rarity and population isolation.

In the Meeman site, measures of clonal diversity varied across the quadrats as some genets were found in only one portion of the sampling area whereas others were more widespread. For example, genet/clone 1 is present in five quadrats spread across the study site, and 52 genets are present only once throughout the site. This finding may be related to the high erodability (providing mobility of ramets during periods of rain) of the loess soil (Memphis silt loams) in which these plants grow at the Meeman site [24]. The rhizomes of *T*. *recurvatum* are generally very weak and easily broken (authors’ observation) and could contribute to the distribution of plants along the hillside. The potential for soil composition and the physical characteristics of the hillside to play a role in population density and structure has previously been noted for this site [17], and as well, rodents could unearth plants and distribute them throughout the site.

We found the flowering stage class to have significantly higher levels of genetic variation when information theory-based calculations were employed whereas traditional population genetic measures demonstrated no detectable differences. The finding that Shannon-Wiener based-measures are more likely to detect differences among means has recently been noted with regard to crop-wild gene flow literature [34–35] and reviewed in Sherwin et al. [36]. Other plant studies of genetic variation in different plant stage classes have demonstrated mixed results with some studies finding no evidence for differences among stage classes (e.g., in another *Trillium* species; [46]) to those demonstrating significant differences (e.g., bamboo saplings had higher levels of genetic diversity than seedlings; [47]). Explanations for these patterns include combinations of random mortality, competition, and inbreeding depression and will require additional studies to further refine which patterns are most influential in *T*. *recurvatum*. Some studies have predicted that genetic variation of genets may be linked to levels of fitness (as measured by reproductive output) [48]. Ramets of *T*. *recurvatum* are difficult to mark in this site, however, future research assessing the reproductive output of genets and levels of genetic variation could yield insight into these hypotheses. Finally, when compared to the other stage classes, the flowering stage generally comprises the fewest numbers of individuals across years [17]; we hypothesize that if flowering adults continue to remain in the minority and/or decline further, future genetic studies would reveal decreasing genetic variation within this class and perhaps at the site overall.

We found no evidence for a correlation between spatial distance of stage classes and genetic distances as might be expected, for example, if seed dispersal was restricted and seeds were dispersed in clusters. However, spatial genetic structure analyses of genets as assessed by *Sp* statistics (which represents the rate of decrease in pairwise kinship with distance) demonstrated significant spatial genetic structure. Our estimate of the *Sp* statistic based upon the logarithm distance slope (*Sp* = 0.0271) is comparable with the predominately outcrossing *Trillium* species, *T*. *grandiflorum* (*Sp* = 0.02494) and can be placed in context with other plant species falling between the mixed mating and outcrossing means of species [22]. The pattern of genets across the Meeman site can also provide information about dispersal patterns [16]. The degree of spatial genetic structure when compared to other animal dispersed species is much greater in our study and even higher than the *Sp* values for gravity dispersed seeds. The clonal nature of *T*. *recurvatum* likely adds to the structuring of genetic variation. The related clonal species *Paris quadrifolia* also demonstrated strong spatial genetic structure of genets [49].

When compared to two other nearby populations of *T*. *recurvatum* in Shelby County, the Meeman site displayed comparable levels of genetic variation and inbreeding (all mean values being positive). The among population structure when all populations were considered was very low, though significantly different from zero. The sampled sites in Shelby County, fall within the central distribution of the range for *T*. *recurvatum* and this species is relatively common with likely additional populations intervening and connecting our populations. At the northern and southern edges of the range, the species is considered rare and of concern; additional genetic studies of those edge populations would be valuable to understanding the population dynamics of this clonal species.

Our results provide novel insights into mating system in *T*. *recurvatum* at the Meeman site. The prevailing view based upon field-based surveys indicated that the majority of recruitment was occurring via asexual production. Our findings of moderate levels of genetic variation demonstrate sexual reproduction is relatively common, and population structure measures indicate that a substantial amount of gene flow, e.g., via seed or pollen, occurs amongst populations. We surveyed sites that were approximately 30 km apart, and a sampling of natural populations in the intervening areas would be beneficial for understanding gene flow at a finer scale. Our nuclear makers suggest a high level of gene flow; however, the use of plastid markers would also be beneficial in this system to assess the relative contribution of seed vs. pollen mediated dispersal for *T*. *recurvatum*. As mentioned previously, highly clonal populations often display negative values of F; and our finding here of positive F coefficients for all three sites studied perhaps indicates biparental inbreeding in the Meeman sight (given the self-incompatibility in the species).

In closing, we report that *T*. *recurvatum* at the focal Meeman site displays higher levels of sexual reproduction than were previously suggested [17]. We also acknowledge that one of the most limiting factors in delimiting genets in a population is the marker number and polymorphism and value the concerns and suggestions of Arnaud-Haond et al. [50] who call for standardizing analyses across clonal studies. We aimed here to follow some of their recommendations for analyzing the discriminatory power of a set of loci. We show that using as few as five population genetic markers revealed spatial genetic structure across the site and will provide future researchers studying this species with genetic markers. Given the spatial genetic structure in the Meeman site, an assessment other populations of *T*. *recurvatum*, especially at the ranges of the species where it is considered rare, would be of value to understanding the population dynamics of this species. Our findings also suggest that other ecological genetic studies may benefit from analyzing data in an information theory framework; for example, in the related species, *T*. *maculatum*, where no evidence for genetic diversity differences among stage classes was reported [46], a reanalysis of the data using the more sensitive measures of information theory of clonal diversity may indeed reveal genetic differences. In addition, since it is often noted that the effects of demographic processes may be realized in a population more quickly than genetic effects, we aim to survey population genetic variation at the Meeman site in the future to assess any changes in levels of variation or spatial patterns of diversity. Finally, comprehensive reconstruction of the population dynamics for clonal, perennial species requires both genetic and long-term demographic data, and the clonal and genetic diversity results presented here provide the necessary inputs for future studies addressing population dynamics questions in *T*. *recurvatum*.

## Supporting information

S1 Fig Microsatellite resolution box plot. Box plot describing the genotypic resolution of microsatellites in the data set.
(TIF)

S1 Table Microsatellite PCR primer sequences and reaction details. Primer sequences and PCR reaction conditions for the newly developed microsatellite markers.
(XLSX)

S2 Table MICRO-CHECKER data. Results of MICRO-CHECKER.
(XLSX)

S3 Table Clonal and population genetic details. This table provides number of different alleles, number of effective alleles, observed heterozygosity, expected heterozygosity, and unbiased (corrected) expected heterozygosity per population for each locus per population and for each quadrat surveyed.
(XLSX)

S4 Table Genet/Clone coordinates. This table provides coordinates of each sampled plant (81 genets) given within quadrats.
(XLSX)

S5 Table Data for clonal occupancy. This table provides the raw data for Fig 1.
(XLSX)

S6 Table Raw microsatellite data. This table provides the genotype scores for the **m**icrosatellite data.
(XLSX)

## References

[1] Tiffney BH, Niklas KJ. Clonal Growth in land plants: a paleobotanical perspective In Population biology and the evolution of clonal organisms. Edited by Jackson J.B.C., Buss L.W., and Cook R.E. Yale University Press, New Haven, CT 1985.

[2] Billings WD, Mooney HA. The ecology of arctic and alpine plants. Biol Rev. 1968;43: 481–529. 10.1111/j.1469-185X.1968.tb00968.x

[3] Cook RE. Growth and development in clonal plant populations In: Population Biology of Clonal Organisms. Edited by Jackson J.B.C., Buss L.W. and Cook R.E.. Yale University Press, New Haven, Connecticut, USA pp. 259–296.1985.

[4] Klimes L, Klimesova J, Hendricks R, van Groenendal JM. Clonal plant architecture: a comparative analysis of form and function In The ecology and evolution of clonal plants. Edited by de Kroon H., and van Groenendal JM. Leiden: Backhuys 1997.

[5] Honnay O, Jacquemyn H. A meta-analysis of the relation between mating system, growth form and genotypic diversity in clonal plant species. Evol Ecol. 2008;22: 299–312. 10.1007/s10682-007-9202-8

[6] Pluess AR, Stöcklin J. Population genetic diversity of the clonal plant Geum reptans (Rosaceae) in the Swiss Alps. Am J Bot. 2004;91: 2013–2021. 10.3732/ajb.91.12.2013 21652350

[7] De Witte LC, J. Longevity of clonal plants: why it matters and how to measure it. Annals Bot. 2010;106: 859–870. 10.1093/aob/mcq191 PMC2990663 20880935

[8] Aigner PA. Ecological and genetic effects on demographic processes: pollination, clonality and seed production in Dithyrea maritima. Biol Conserv. 2004;116: 27–34. 10.1016/S0006-3207(03)00170-8

[9] Garnder SN, Mangel M. Modeling investments in seeds, clonal offspring, and translocation in a clonal plant. Ecol. 1999;80: 1202–1220. 10.2307/177068

[10] Silvertown J. The evolutionary maintenance of sexual reproduction: evidence from the ecological distribution of asexual reproduction in clonal plants. Int J Plant Sci. 2008;169: 157–168. 10.1086/523357

[11] Bengtsson BO, Ceplitis A. The balance between sexual and asexual reproduction in plants living in variable environments. J Evol Biol. 2000;13: 415–422. 10.1046/j.1420-9101.2000.00187.x

[12] Ellstrand NC, Roose ML. Patterns of genotypic diversity in clonal plant species. Am J Bot. 1987;74: 123–131.

[13] Widén B, Cronberg N, Widén M. Genotypic diversity, molecular markers and spatial distribution of genets in clonal plants, a literature survey. Folia Geobotanica. 1994;29: 245–263. 10.1007/BF02803799

[14] Vekemans X, Hardy OJ. New insights from fine-scale spatial genetic structure analyses in plant populations. Mol Ecol. 2014;13: 921–935. 10.1046/j.1365-294x.2004.02076.x 15012766

[15] Harada Y, Iwasa Y. Analyses of Spatial Patterns and Population Processes of Clonal Plants. Res Popul Ecol. 1996;38: 153–164.

[16] Sawyer NW. Reproductive ecology of Trillium recurvatum (Trilliaceae) in Wisconsin. Am Mid Nat. 2010;163: 146–160. 10.1674/0003-0031-163.1.146

[17] Moore JE, Franklin SB, Weins G, Collins BS. Long-term demography of Trillium recurvatum (Beck) on loess bluffs in western TN. AoB PLANTS. 2012; 10.1093/aobpla/pls015 PMC3357055 22616024

[18] Knight TM. The effects of herbivory and pollen limitation on a declining population of Trillium grandiflorum. Ecol Appl. 2004;14: 915–928.

[19] Leege LM, Thompson JS, Parris DJ. The responses of rare common trilliums (Trillium reliquum, T. cuneatum, and T. maculatum) to deer herbivory and invasive honeysuckle removal. Castanea. 2010;75: 433–443. 10.2179/09-048.1

[20] Wagenius S, Londsdorf E, Neuhauser C. Patch aging and the S-allee effect: breeding system effects on the demographic response of plants to habitat fragmentation. Am Nat. 2007;169: 383–397. 10.1086/511313 17230399

[21] O’Connor RP. Special Plant Abstract for Trillium recurvatum (prairie trillium) Lansing, MI: Michigan Natural Features Inventory 2007.

[22] Kalisz S, Hanzawa FM, Tonsor SJ, Thiede DA, Voigt S. Ant-mediated seed dispersal alters pattern of relatedness in a population of Trillium grandiflorum. Ecol. 1999;80: 2620–2634.

[23] Case FW, Case RB. Trilliums. Timber Press, Portland, OR 1997.

[24] McCarthy KP. An analysis of gully development in Meeman-Shelby Forest State Park, Tennessee. MSc thesis, Department of Geography, The University of Memphis, Memphis, TN. 1990.

[25] Hamilton MB, Pincus EL, Di-Fiore A, Fleischer RC. Universal linker and ligation procedures for construction of genomic DNA libraries enriched for microsatellites. Biotechniques. 1999;27:500–507. 10.2144/99273st03 10489609

[26] Pellicer J, Kelly LJ, Leitch IJ, Zomlefer WB, Fay MF. A universe of dwarfs and giants: genome size and chromosome evolution in the monocot family Melanthiaceae. New Phytologist. 2014;201:1484–1497. 10.1111/nph.12617 24299166

[27] Faircloth B.C., 2008 MSATCOMMANDER: Detection of microsatellite repeat arrays and automated, locus‐specific primer design. Molecular ecology resources, 8(1), pp.92–94. 10.1111/j.1471-8286.2007.01884.x 21585724

[28] Schuelke M. An economic method for the fluorescent labeling of PCR fragments. Nat Biotechnol. 2000;18:233–234. 10.1038/72708 10657137

[29] Van Oosterhout C, Hutchinson WF, Wills DP, Shipley P. MICRO-CHECKER: software for identifying and correcting genotyping errors in microsatellite data. Mol Ecol Notes. 2004;4: 535–538.

[30] Mandel JR. Clonal diversity, spatial dynamics, and small genetic population size in the rare sunflower, Helianthus verticillatus. Conserv Genet. 2010;11: 2055–2059. 10.1007/s10592-010-0062-3

[31] Bentley KE, Mauricio R. High degree of clonal reproduction and lack of large-scale geographic patterning mark the introduced range of the invasive vine, kudzu (Pueraria montana var. lobate), in North America. Am J Bot. 2016;103: 1499–1507. 10.3732/ajb.1500434 27555435

[32] Meirmans PG, Van Tienderen PH. GENOTYPE and GENODIVE: two programs for the analysis of genetic diversity of asexual organisms. Mol Ecol Resources. 2004;4: 792–794. 10.1111/j.1471-8286.2004.00770.x

[33] Peakall E, Smouse PR. GenAlEx 6.5: genetic analysis in Excel. Population genetic software for teaching and research. Bioinformatics. 2012;28: 1367–4803. 10.1093/bioinformatics/bts460 PMC3463245 22820204

[34] Campbell LG, Lee D, Shukla K, Waite TA, Bartsch D. An ecological approach to measuring the evolutionary consequences of gene flow from crops to wild or weedy relatives. Appl Plant Sci. 2016;4: 1500114 10.3732/apps.1500114 PMC4795919 27011898

[35] Mandel JR, Ramsey AJ, Iorizzo M, Simon PW. Patterns of gene flow between crop and wild carrot, Daucus carota (Apiaceae) in the United States. PLoS ONE. 2016; e0161971 10.1371/journal.pone.0161971 27603516 PMC5014312

[36] Sherwin WB, Chao A, Jost L, Smouse PE. Information Theory Broadens the Spectrum of Molecular Ecology and Evolution. Trends Ecol Evol. 2017;32: 948–63. 10.1016/j.tree.2017.09.012 29126564

[37] Nei M. Estimation of average heterozygosity and genetic distance from a small number of individuals. Genetics. 1978;89: 583–590. 17248844 10.1093/genetics/89.3.583 PMC1213855

[38] Kamvar ZN, Tabima JF, Grünwald NJ. Poppr: an R package for genetic analysis of populations with clonal, partially clonal, and/or sexual reproduction. 2014; PeerJ 2:e281 10.7717/peerj.281 24688859 PMC3961149

[39] R Core Team. R: A Language and Environment for Statistical Computing Vienna, Austria: R Foundation for Statistical Computing 2016;Version 3.3.2.

[40] Mantel N. The detection of disease clustering and a generalized regression approach. Cancer Res. 1967;27: 209–220. 6018555

[41] Hardy OJ, Vekemans X. SPAGeDI: A versatile computer program to analyse spatial 6 genetic structure at the individual or population levels. Mol Ecol Notes. 2002; 2: 618–620.

[42] Halkett F, Simon JC, Balloux F. Tackling the population genetics of clonal and partially clonal organisms. Tren Ecol & Evol. 2005;20: 194–201. 10.1016/j.tree.2005.01.001 16701368

[43] Gaudeul M, Delahaye T, Muller S. AFLP markers show low levels of clonal propagation and high genotypic diversity in the rare, southernmost populations of Linnaea borealis L.(Caprifoliaceae) in the Western Alps. Genetica. 2019;147: 79–90. 10.1007/s10709-019-00054-6 30767171

[44] Gonzales E, Hamrick JL, Smouse PE. Comparison of clonal diversity in mountain and Piedmont populations of Trillium cuneatum (Melanthiaceae‐Trilliaceae), a forest understory species. Am J Bot. 2008;95: 1254–1261. 10.3732/ajb.2007159 21632330

[45] Gonzales E, Hamrick JL. Distribution of genetic diversity among disjunct populations of the rare forest understory herb, Trillium reliquum. Heredity. 2005;95: 306 10.1038/sj.hdy.6800719 16094302

[46] Walker AN, Foré SA, Collins BS. Fine-scale structure patterning of Trillium maculatum (Liliaceae) population. Botany. 2009; 87: 223–230. 10.1139/Bo8-135

[47] Abreu AG, Grombone-Guaratini MT, Moreira T. Genetic diversity and age class structure of seedings and saplings after a mast flowering of bamboo in the Brazilian Atlantic Forest. Int J Plant Sci. 2014;175: 319–327. 10.1086/674448

[48] Gargiulo R, Ilves A, Kaart T, Fay MF, Kull T. High genetic diversity in a threatened clonal species, Cypripedium calceolus (Orchidaceae), enables long-term stability of the species in different biogeographical regions in Estonia. Bot J Linn Soc. 2018;186: 560–571.

[49] Jacquemyn H, Brys R, Honnay O, Hermy M, ROLDÁN‐RUIZ I. Local forest environment largely affects below‐ground growth, clonal diversity and fine‐scale spatial genetic structure in the temperate deciduous forest herb Paris quadrifolia. Mol Ecol. 2005;14: 4479–4488. 10.1111/j.1365-294X.2005.02741.x 16313608

[50] Arnaud‐Haond S, Duarte CM, Alberto F, Serrao EA. Standardizing methods to address clonality in population studies. Mol Ecol. 2007;16: 5115–5139. 10.1111/j.1365-294X.2007.03535.x 17944846

