# Supplementary figures and images for: Clonal diversity and spatial genetic structure in the long-lived herb, Prairie trillium
Source: PLoS One. 2019 Oct 21;14(10):e0224123. doi: 10.1371/journal.pone.0224123 (PMC6802849; doi:10.1371/journal.pone.0224123)

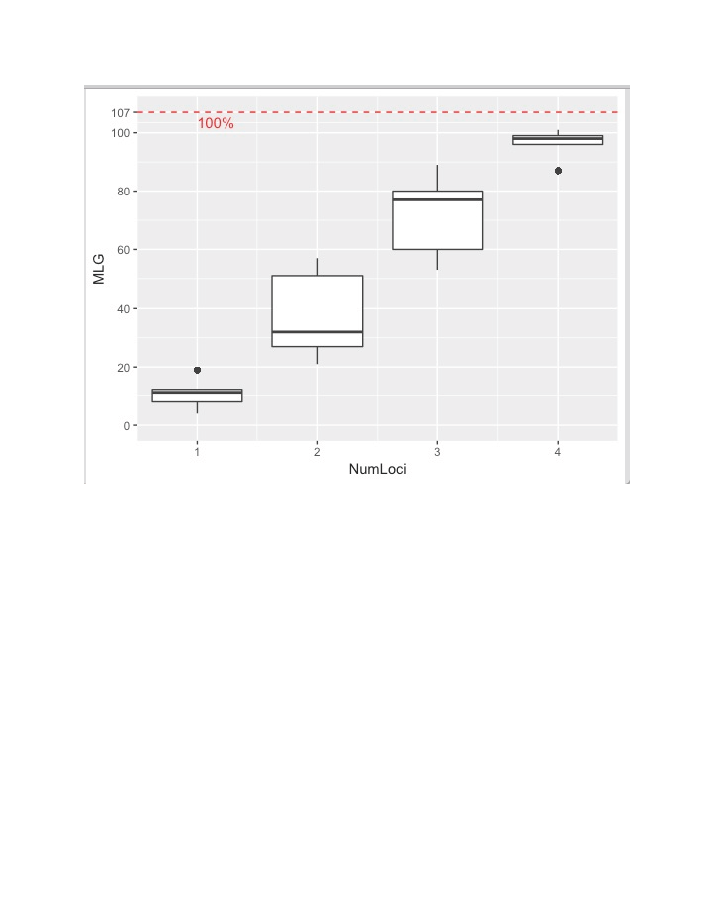

Supplement: S1 Fig — Box plot describing the genotypic resolution of microsatellites in the data set. (TIF) [file pone.0224123.s001.tif]
